# Aflatoxin B1 Toxicity and Protective Effects of Curcumin: Molecular Mechanisms and Clinical Implications

**DOI:** 10.3390/antiox11102031

**Published:** 2022-10-14

**Authors:** Chongshan Dai, Erjie Tian, Zhihui Hao, Shusheng Tang, Zhanhui Wang, Gaurav Sharma, Haiyang Jiang, Jianzhong Shen

**Affiliations:** 1 Department of Veterinary Pharmacology and Toxicology, College of Veterinary Medicine, China Agricultural University, Beijing 100193, China; 2 Key Biology Laboratory of Chinese Veterinary Medicine, Ministry of Agriculture and Rural Affairs, Beijing 100193, China; 3 College of Animal Science and Technology, Henan University of Science and Technology, Luoyang 471023, China; 4 Advanced Imaging Research Center, University of Texas Southwestern Medical Center, Dallas, TX 75390, USA

**Keywords:** AFB1, toxicity, detoxification effects, curcumin, molecular mechanisms

## Abstract

One of the most significant classes of mycotoxins, aflatoxins (AFTs), can cause a variety of detrimental outcomes, including cancer, hepatitis, aberrant mutations, and reproductive issues. Among the 21 identified AFTs, aflatoxin B1 (AFB1) is the most harmful to humans and animals. The mechanisms of AFB1-induced toxicity are connected to the generation of excess reactive oxygen species (ROS), upregulation of CYP450 activities, oxidative stress, lipid peroxidation, apoptosis, mitochondrial dysfunction, autophagy, necrosis, and inflammatory response. Several signaling pathways, including p53, PI3K/Akt/mTOR, Nrf2/ARE, NF-κB, NLRP3, MAPKs, and Wnt/β-catenin have been shown to contribute to AFB1-mediated toxic effects in mammalian cells. Curcumin, a natural product with multiple therapeutic activities (e.g., anti-inflammatory, antioxidant, anticancer, and immunoregulation activities), could revise AFB1-induced harmful effects by targeting these pathways. Therefore, the potential therapeutic use of curcumin against AFB1-related side effects and the underlying molecular mechanisms are summarized. This review, in our opinion, advances significant knowledge, sparks larger discussions, and drives additional improvements in the hazardous examination of AFTs and detoxifying the application of curcumin.

## 1. Introduction

Mycotoxins are naturally occurring toxins and secondary metabolites that are mostly produced by fungi of the genera *Penicillium*, *Aspergillus*, *Claviceps*, *Fusarium,* and *Alternaria,* which contaminate basic food products worldwide [1]. There have been over 100,000 fungi discovered to date, and over 500 mycotoxins have been linked to cardiovascular disease, immunological dysregulation, chronic liver disease, renal disease, cancer, and neurological disorders in both humans and animals [2,3,4,5]. In recent decades, mycotoxin contaminations (e.g., aflatoxins, fumonisins, ochratoxins, zearalenone, trichothecenes, patulin, citrinin, ergot alkaloids, and tremorgenic toxins) could be detected in a variety of grains and foods, including in cereals (e.g., maize, corn, wheat, rice, sorghum, and millet), spices (e.g., black pepper, paprika, chili, turmeric, and ginger), nuts (e.g., walnuts, peanuts, pistachios, Brazil nut, and coconut), tea, drinking water, seeds, Chinese herbal medicine, and beer, which has raised concerns worldwide [1,6,7].

Aflatoxins (AFTs), one of the most significant classes of mycotoxins, were primarily generated by numerous fungal species, including *Aspergillus* (A.) *flavus*, *A. nomius*, and *A. parasiticus* [8]. With the advancement of analytical and detection techniques, approximately 21 AFTs have been identified, including aflatoxin B1 (AFB1), AFB2, AFB3, aflatoxin B2a (AFB2a), aflatoxin G1 (AFG1), AFG2, AFG2a, aflatoxin B1-8,9-epoxide (AFBO), aflatoxin P1 (AFP1), aflatoxicol M1 (AFM1), AFM2, AFM2a, AFGM1, AFGM2, AFGM2a, aflatoxin Q1 (AFQ1), aflatoxicol H1(AFH1), aflatoxicol (AFL), parasiticol, aflatrem, and aspertoxin [9,10]. The primary four are AFB1, AFB2, AFG1, and AFG2, with the order of toxicity being AFB1 > AFG1 > AFB2 > AFG2 [8,10]. AFB1 could induce hepatotoxicity, nephrotoxicity, immunotoxicity, and reproductive toxicity in vitro and animal models [2,3,4,5]. A recent study found that a low dose of AFB1 exposure also caused a decrease in locomotor activity and anxiety with pathologic damage in brain tissues, indicating neurotoxicity [11]. The International Agency for Research on Cancer (IARC) has classified AFB1 and AFM1 as group 1 and group 2B human carcinogens, respectively [12,13]. Hepatocellular carcinoma (HCC) is a well-established adverse effect of AFTs exposure, and it is reported that about 4.6% to 28.2% of all global incidences of HCC are positively related to AFTs exposure [14]. The majority of HCC occurs in several countries in sub-Saharan Africa and southeast Asia, which usually have a high incidence of Hepatitis B infection, as well as significant levels of AFTs exposure in food [14]. Over the last 10 years, the occurrence of AFB1 in food commodities is still high in several countries (including China, India, and Korea), and the positive rates of AFB1 in rice and wheat samples are both about 90%–100% [9]. In a recent retrospective study of the disease caused by dietary exposure to AFTs in China from 2010 to 2020, Chen and colleagues found that the total amount of AFTs consumed by Chinese people through corn, peanut, and their oil products was high, at 4.018 ng/kg body weight (BW)/day, leading to 1.53 additional cases of hepatocellular carcinoma per 100,000 people per year [15]. Due to its elevated risk level and toxicity, scientists from all around the world have been investigating the molecular pathways and detoxification approaches linked to AFB1 toxicity [9,16].

In order to avoid AFT contamination and complete detoxification, it is essential to comprehend the synthesis, toxicity, and molecular mechanism of AFTs. Several natural products or active compounds, including lycopene, grape seed proanthocyanidin extract, fucoidan, sodium selenite, beta-1,3-glucan, selenium, and sporoderm-broken ganderma lucidum spores, have been found to mitigate AFB1-exposure-induced organ damage [17,18,19,20,21,22]. Curcumin (1,7-bis (4-hydroxy-3-methoxyphenyl)-1,6-heptadiene-3,5-dione) is a polyphenolic molecule extracted from the *Curcuma longa* (turmeric), which has been shown to have anti-inflammatory, anticancer, antioxidative stress, and immunological modulating characteristics [23,24]. Animal experimental or in vitro studies had reported that curcumin supplementation could effectively reduce AFB1-induced liver damage, renal dysfunction, and intestinal damage via inhibiting oxidative stress, apoptosis, inflammation, necrosis, and CYP450 enzyme expression [25,26,27,28,29,30,31,32,33,34]. In addition, several studies also reported that curcumin in combination with black tea could synergistically improve AFB1-induced liver and kidney damage [35]. Importantly, human clinical trials or animal experiments have shown that curcumin is safe and tolerable [23]. The toxic effects, molecular processes, and detoxifying properties of curcumin are discussed in this review. It also discusses the possible therapeutic benefits of curcumin as a detoxifying agent. Our goal is that this review will be insightful and spur more investigation into curcumin’s potential as a detoxifying agent against AFTs.

## 2. An Overview of AFB1-Induced Toxic Effects and Molecular Mechanisms

AFB1 and its metabolites also exhibit a number of toxic adverse effects, such as liver, kidney, spleen, brain, gut, skin, testis, and cardiac tissue toxicity, as well as mutagenicity, teratogenicity, and carcinogenicity [19,20,36,37,38,39,40,41]. Importantly, the liver is the main target organ because the vast majority of AFB1 is metabolized by the liver. Therefore, AFB1 exposure has been linked to hepatocarcinogenesis in humans, as well as in other animals, including birds, fish, rodents, and nonhuman primates [42]. Additionally, AFB1 might greatly inhibit the immune response, raising the risk of cirrhosis and HCC in those with chronic hepatitis B virus infection [43,44]. Excess reactive oxygen species (ROS) production, DNA damage, oxidative stress, lipid peroxidation, apoptosis, mitochondrial dysfunction, autophagy, necrosis, and inflammatory response were all implicated in the pathways of AFB1-induced cytotoxicity or cell death [45,46,47,48,49,50]. A number of signaling pathways, such as those involving p53, p21, phosphatidylinositide 3-kinase (PI3K), protein kinase B (PKB, also known as Akt), mammalian target of rapamycin (mTOR), NF-E2-related factor 2 (Nrf2), antioxidant responsive element (ARE), nuclear factor kappa-B (NF-κB), Toll-like receptor 4 (TLR4), TLR2, NOD-like receptor thermal protein domain associated protein 3 (NLRP3)/Caspase-1, mitogen-activated protein kinases (MAPKs), and Wnt/β-catenin pathways, were exemplified to participate in AFB1-induced toxic effects in in vitro and in vivo models [45,46,47,48,49,50]. As a result, these pathways represent crucial targets for preventing or treating AFB1-induced harmful consequences in both animals and humans.

## 3. Biological Properties of Curcumin

**Figure 1:**
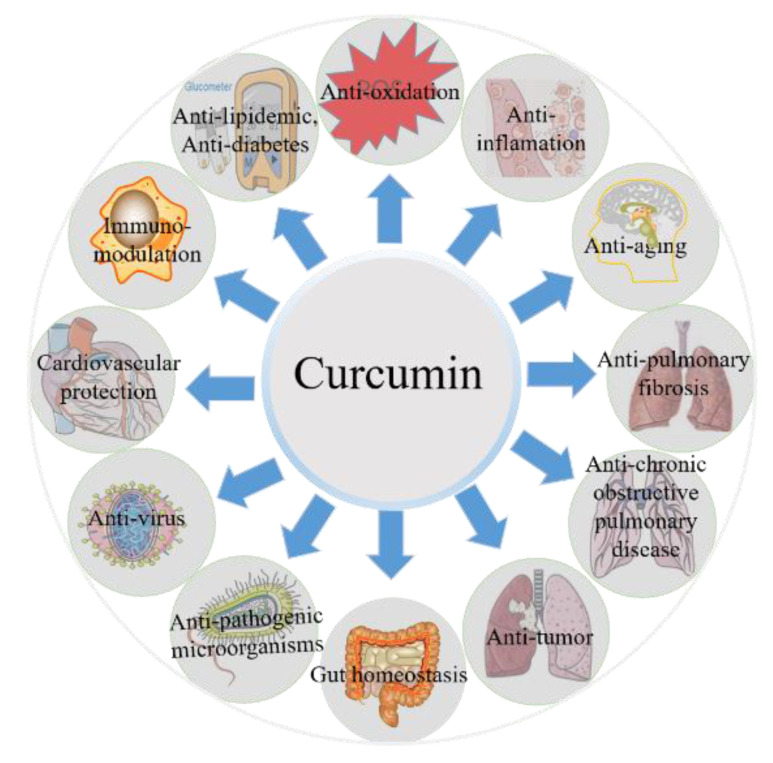
A diagram depicting the biological effects of curcumin.

Curcumin is the main active agent of the root extract of Curcuma longa (turmeric), a plant extensively produced in India, China, and many other Asian countries [51]. In commercially available curcumin powder, there are three primary components, namely diferuloylmethane (i.e., curcumin; at 82%) and its derivatives demethoxycurcumin (i.e., DMC; at 15%) and bisdemethoxycurcumin (i.e., BDMC; at 3%), respectively [52]. The significant biochemical and biological effects of curcumin include antioxidation, anti-inflammation, anti-lipidemia, antiviral, antibacterial, anticancer, immunomodulation, cardiovascular protection, and neuroprotection (Figure 1) [23,53,54,55,56,57,58,59]. Curcumin also had substantial antioxidative effects on protein carbonylation, lipid peroxidation, free radical production, and mitochondrial permeability transition, ultimately preventing multiple kinds of cell death, including pyroptosis, ferroptosis, apoptosis, and necroptosis [57]. Curcumin has been shown to target many signaling pathways, including AMPK, p53, p21, AKT, mTOR, Nrf2/ARE, NF-κB, NLRP3, MAPKs, c-JUN, peroxisome-proliferator-activated receptor (PPAR), and Wnt/-catenin pathways, ultimately inhibiting nephrotoxicity, neurotoxicity, hepatotoxicity, immunotoxicity, lung damage, and blood toxicity caused by bio-toxins (e.g., ochratoxin A, fumonisin B1, and deoxynivalenol), antibiotic and anticancer drugs (e.g., colistin, cisplatin, vancomycin, and gentamicin, etc.), heavy metals (e.g., copper, lead, arsenic, cadmium, chromium, and mercury, etc.), and pathogenic pathogens (e.g., *Staphylococcus aureus*, *Enteropathogenic Escherichia coli*, and *Mycobacterium tuberculosis*) [54,60,61,62,63,64,65,66,67]. Additionally, several clinical studies have shown that curcumin has a strong therapeutic potential for treating a few chronic illnesses, including pulmonary, cancer, neurological, cardiovascular, infectious, neoplastic, psychiatric, and metabolic disorders [68]. Not surprisingly, several in vitro and animal experimental studies confirmed that curcumin supplementation could offer strong protective effects against AFB1-induced liver injury, mutagenicity, hepatocarcinogenicity, renal dysfunction, ileum damage, immune toxicity via regulating multiple above-mentioned targets [26,34,60,69,70,71,72,73,74,75,76]. The detailed molecular pathways are discussed in the section below.

## 4. Curcumin’s Protective Role in Preventing AFB1-Induced Toxicity and the Potential Molecular Mechanisms

Although there have been some natural products that can improve the toxicity induced by AFB1, curcumin is the most typical representative based on its lower cost and higher safety. A summary of in vitro and in vivo studies involving the protective effects of curcumin or curcuminoids on AFB1-induced toxicity is shown in Table 1. The underlying molecular mechanism is addressed, mainly involving the inhibition of oxidative stress, blockade of the inflammatory response, inhibition of apoptosis, downregulation of necroptosis, promotion of autophagy, regulation of immune response, and intervention of AFB1′s metabolism.

### 4.1. AFB1 Exposure Induces Oxidative Stress and the Inhibitory Effect of Curcumin

Excess ROS generation exceeds the ability of the intracellular antioxidant system to scavenge free radicals, resulting in oxidative stress [88]. Oxidative stress is one of the critical molecular mechanisms of adverse effects caused by many toxic chemical hazards or drugs (e.g., heavy metals, mycotoxins, pesticides, chemotherapy drugs, and antibiotics) [88]. Multiple studies reported that AFB1 and its derivatives could trigger oxidative stress by inducing the formation of ROS or decreasing intracellular antioxidants or the activities of antioxidant enzymes [36,70,73,89,90,91,92,93,94,95,96,97,98]. In the liver tissues, the cytochrome (CYP)1A2 and CYP3A4 could bio-transform into AFBO, which is a harmful and extremely active, electrophilic metabolite. It has been reported that AFBO could also induce the formation of ROS and oxidative stress [9]. Additionally, the hepatic glutathione S-transferase system was able to detoxify AFBO by glutathione (GSH) conjugation, resulting in the formation of their non-toxic polar versions, i.e., AFBO-GSH [9]. Recently, in a rat model, Abdel-Daim et al. reported that acute exposure to AFB1 at a dose of 50 μg/kg BW (via intraperitoneal injection) for 14 days significantly increased malondialdehyde (MDA) and nitric oxide (NO) levels and decreased SOD, CAT, and GPX activities and reduced GSH content in liver tissues, followed by induction of oxidative stress [22]. Low-dose exposure to AFB1 also causes a decrease in GPX activities and GSH levels in the brain tissues, followed by induction of a decrease in locomotor activity, anxiety, and neurotoxicity in rats [11].

Wang et al. discovered that oral administration of AFB1 at 750 μg/kg BW for 30 days might produce significant kidney oxidative damage, as demonstrated by the loss of antioxidative enzyme (e.g., CAT and SOD) activities and the decreases in GSH levels and increases in hydrogen peroxide (H_2_O_2_) and levels of MDA in the kidney tissues; meanwhile, oral administration of curcumin supplementation could significantly inhibit AFB1-induced oxidative stress by upregulating the GSH content and SOD and CAT activities [70]. Consistently, curcumin supplementation could also significantly improve AFB1-exposure-induced cytotoxicity in bovine fetal hepatocyte-derived cells, renal damage in mice and chickens, and liver, spleen, and ileum damage in ducks by inhibiting the production of ROS and oxidative stress through the upregulation of activities of antioxidant enzymes and levels of antioxidants or free radical scavenging [60,69,70,72,74,75]. Very recently, Damiano et al. found that curcumin supplementation significantly inhibits the expression of NADPH Oxidase 4 (NOX4) mRNA and protein, thus inhibiting AFB1-induced renal oxidative stress in chicken [75]. NOX4 is the main productor of endogenous ROS production [99,100]. Our recent study found that inhibiting NOX4 expression might considerably reduce colistin-induced nephrotoxicity and lung damage [101,102]. Curcumin treatment also dramatically alleviated oxidative stress damage in seminal vesicles by regulation of NOX1, NOX2, and NOX4 expressions [103]. NOX4 is the critical mediator in the process of TGF-β-induced inflammatory response and fibrotic response [104]. The evidence indicated that NOX4 may play a critical role in the protective effect of curcumin against AFB1-induced oxidative stress and inflammatory response. The precise molecular mechanisms still need more investigations.

In response to oxidative damage, Nrf2 is a crucial transcription factor [105]. More than 200 genes involved in the processes of antioxidant, anti-inflammatory, and xenobiotic metabolism may be produced as a result of Nrf2 activation [106]. Under a normal physiological microenvironment, Nrf2 usually locates in the cytoplasm and interacts with Kelch-like ECH-associated protein 1 (Keap1), an adaptor protein that connects the Nrf2 and Cullin3-Rbx1 E3 ligase (CULLIN3) [106]. Keap1 could aid in the ubiquitination and subsequent destruction of Nrf2 by 26S proteasomes in the cytoplasm [107]. Under stressed environments, however, the ability of CULLIN3 to ubiquitinate Nrf2 is suppressed, which allows Nrf2 to be transcribed into the nucleus, inducing the expression of the protective genes in response to various stresses [106]. AFB1 exposure inhibited the expression of Nrf2 and its downstream proteins, including heme oxygenase-1 (HO-1), quinone oxidoreductase 1 (NQO1), and glutamate-cysteine ligase catalytic subunit (GCLC), causing oxidative stress, liver damage, and nephrotoxicity in mice and broiler chicks [48,70,95]. It is well documented that curcumin is an inducer of Nrf2 [107]. Curcumin could directly bind to the cysteine 151 (Cys151) site of Keap1 protein and promote the liberation of Nrf2 [107]. Curcumin supplementation may greatly increase the expression and transcriptional activity of Nrf2, therefore reducing AFB1-induced tissue damage in the liver, kidney, and ileum [69,70,71,79]. Caveolin-1 is a major multifunctional scaffolding protein, and it could serve as a negative or positive modulator of cell signaling pathways, including PI3K/Akt and Nrf2 pathways [108]. The possibility of targeting caveolin-1 has been raised [109]. A recent study showed that caveolin-1 knockdown could significantly decrease AFB1-induced oxidative stress, apoptosis, and hepatotoxicity by decreasing the interaction of Keap1 and Nrf2 in the cytosol and promoting the transcriptional activity of Nrf2 [108]. This finding also contributed to explaining the molecular mechanisms of curcumin’s protective effects.

**Figure 2:**
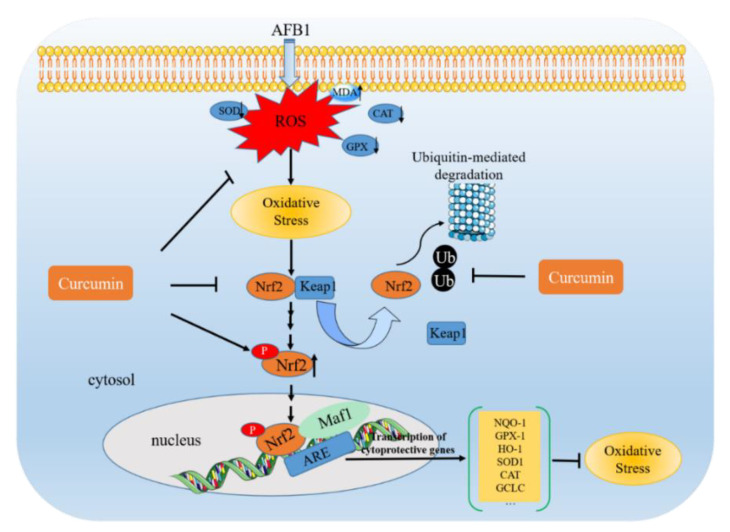
Curcumin improves AFB1-induced toxicity via inhibiting the production of ROS and oxidative stress and activating the Nrf2-mediated antioxidant defense system.

In summary, as shown in Figure 2, curcumin supplementation could protect against AFB1-induced oxidative stress in various tissues by directly scavenging the free radical, upregulating the intracellular antioxidant enzymes’ activities, and activating the Nrf2 pathway.

### 4.2. AFB1 Exposure Causes Immunosuppression, Inflammatory Response, Necroptosis, and the Regulation of Curcumin

Chronic AFB1 exposure may inhibit cell-mediated immunity and lymphoblastogenesis, cause delayed skin hypersensitivity, lose the graft-versus-host response, decrease the number of CD4 cells produced in the spleen, and increase the levels of heat-stable serum phagocytosis factors, all of which may contribute to the development of various inflammation-related diseases, such as HCC and inflammatory bowel disease [36,110,111,112,113]. Because of the immunosuppression, AFB1 exposure generally makes host cells more susceptible to infections caused by hepatitis B and C viruses, as well as the swine influenza virus [114,115,116,117]. Previous studies revealed that NF-κB, TLR4, AHR, NRLP3, and receptor-interacting serine/threonine kinase 1 (RIPK1)/RIPK3/mixed lineage kinase domain-like pseudokinase (MLKL) pathways are involved in the inflammatory response, immunotoxicity, and necroptosis, which is brought on by AFB1 [113]. The detailed molecular mechanisms are addressed below.

The activation of NF-κB involved the degradation of inhibitor kappa B alpha (IκBα) or its phosphorylation [118,119,120], and it mediated the expression of more than 500 genes, such as TNF-α, IL-1β, IL-10, and IL-6 [121]. AFB1 exposure at a low dose by diet supplementation could upregulate the expression of NF-κB, IκB-α, IL-6, TNF-α, IL-1β, and chemokine CCL20 mRNAs, followed by triggering a marked inflammatory response in the liver tissues [122]. Consistently, Long et al. found that in mice that were intragastrically administered with AFB1 at a dose of 100 μg/kg BW for six weeks, significantly increased TNF-α, IFN-γ, IL-1β, and IL-6 protein expression in the serum and mRNA expression in the spleen tissue were detected, indicating an induction of immune toxicity [18]. These findings further confirmed that the activation of the NF-κB signaling pathway may play a critical role in AFB1-induced inflammatory response.

Pathogen-derived substances (e.g., lipopolysaccharide (LPS)), especially those from Gram-negative bacteria, can be identified by Toll-like receptor 4 (TLR4), a pattern recognition receptor (PRR) [123]. TLR4 activation could trigger transcription factor NF-κB-mediated inflammatory response or transcription factor interferon regulatory factor 3 (IRF3)-mediated production of type I interferons that respond to infections caused by most viruses [123]. Of note, TLR4-activation-mediated NF-κB transcriptional activation is required by the MyD88 protein [124]. Recently, Mehrzad’s study found that AFB1 exposure (i.e., 10 ng/mL for 12 h) could significantly upregulate the expression of MyD88, NF-κB, tumor necrosis factor-alpha (TNF-α), TLR2, TLR4, cyclooxygenase 2 (COX-2), human leukocyte antigen–DR isotype (HLA-DR), CD209, CD16, C-C motif chemokine receptor 7 (CCR7), and lymphocyte function-associated antigen 3 (LFA3), and it could significantly downregulate the expression of CD11c and CD64, Aryl hydrocarbon receptor (AhR), and transforming growth factor-β (TGF-β) in human dendritic cells, indicating that AFB1 exposure could alter the transcription of key functional immune and inflammatory genes, phagocytosis, and survival of human dendritic cells [113]. Consistent findings were confirmed in the liver and ileum tissues of mice and liver tissues of chicken [34,49]. Furthermore, AFB1-induced activation of TLR4/NF-κB may be attributed to the production of LPS caused by AFB1-induced gut-microbiota-dysfunction-derived abundance of LPS-producing related bacteria [49]. An early study found that LPS could enhance AFB1-induced liver toxicity, and this was dependent on the production of TNF-α [125]. Although the precise molecular mechanisms remain unclear, the current finding could confirm that gut dysbiosis in humans or animals may exacerbate AFB1-induced toxic effects. This finding greatly facilitates the understanding of the crosstalk between the bacterial toxin and AFTs and the chronic inflammatory effect in gastrointestinal and liver tissues caused by AFB1 at the sub-toxic exposure level.

AFB1 exposure may promote necroptosis in chicken liver tissues by raising the expression of necroptosis-related kinases RIPK1, RIPK3, and MLKL [34]. MLKL activation could promote the formation of plasmalemma channels, thus resulting in membrane disintegration [34]. MLKL is known to be a substrate of RIPK3, which can induce the formation of an NLRP3/caspase-1 inflammasome, as well as promote RIPK3-dependent caspase-8 activation, finally resulting in caspase-8 cleavage of pro-IL-1, and induce both inflammation and necroptotic cell death; this RIPK3 process is not dependent on the presence of MLKL [126]. These data indicated that AFB1-induced necroptosis involved both MLKL-dependent or -independent pathway.

Curcumin has significant immunomodulatory and anti-inflammatory effects and can target various signaling molecules (e.g., NF-κB, COX-2, 5-lipoxygenase, TLR4, IL-6, IL-8, IL-1, and TNF-α) directly or indirectly and immune cells (e.g., macrophages, natural killer cells, neutrophils, mast cells, lymphocytes, and innate lymphoid cells) [127]. Curcumin supplementation substantially decreased the production of TLR4, IL-6, IL-1, IL-8, TNF-α, and NF-κB mRNAs in the hepatocytes of broiler chickens exposed to a low dosage of AFB1 [80]. Consistently, Jin et al. found that curcumin supplementation could significantly improve AFB1-induced inflammatory response via the inhibition of NF-κB, TLR4, and NRLP3 pathways in the ileum tissues of ducks [69]. Pauletto et al. showed that curcumin and its extracts could effectively improve AFB1-exposure-induced inflammatory response in bovine BFH12 cells in vitro [60]. Li et al. found that curcumin supplementation significantly blocks the activation of TLR4/MyD88, followed by inhibiting the activation of NF-κB and its downstream genes’ expression, including iNOS, IL-6, IL-1β, and TNF-α genes [34]. Meanwhile, curcumin supplementation could significantly inhibit the expression of RIPK1, RIPK3, and MLKL, thus improving AFB1-induced necroptosis in the liver tissues of chicken [34]. In accordance with these findings, Wang et al. discovered that curcumin supplementation substantially inhibited AFB1-induced necroptosis in mouse livers by downregulating the expression of Caspase-1, NLRP3, and GSDMD while increasing the expression of antioxidant molecules (i.e., CAT, SOD, HO-1, and NQO1) [71]. These studies indicated that curcumin supplementation could effectively improve AFB1-induced inflammatory response and necroptosis by targeting the TLR4, NF-κB, and NRLP3 pathway. In addition, the ameliorated effects of curcumin on AFB1-induced expression of inflammatory response and immunosuppression may involve the regulation of long non-coding RNA expression [73]. Solis-Cruz et al. found that curcumin supplementation could also improve the immune functions of animals to combat AFB1-induced toxic effects [29]. Moreover, several studies have found that oral curcumin supplementation could rebalance the ratio between beneficial and harmful bacteria in gut microbiota, i.e., upregulating the abundance of beneficial bacteria strains, including *Lactobacilli*, *Bifidobacteria*, and butyrate-producing bacteria, and downregulating the abundance of pathogenic strains, including *Enterobacteria*, *Coriobacterales*, *Prevotellaceae*, and *Rikenellaceae*, and other LPS-producing bacteria [128,129,130]. Indeed, several studies have found that probiotic supplementation could effectively reduce the accumulation of AFB1 in liver tissues and AFB1-induced oxidative damage [131,132]. Therefore, the promising effects of curcumin on gut microbiota may also contribute to the protective effects of curcumin on AFB1-induced chronic inflammatory response. It has been reported that curcumin could protect the intestinal barrier against an inflammatory response in in vitro and animal models [133]. The precise molecular mechanisms are complex and still need further investigation.

**Figure 3:**
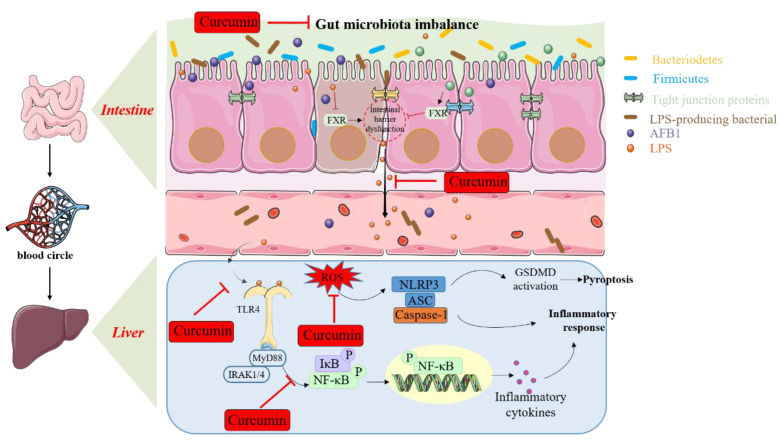
The regulated effects of curcumin on AFB1-induced inflammatory response via modulation of gut microbiota and the inhibition of NF-κB, TLR4, and NRLP3 pathways.

Curcumin supplementation, as demonstrated in Figure 3, may protect against AFB1-induced immunosuppression, inflammatory response, and necroptosis, which may involve the inhibition of the NF-κB, TLR4, and NRLP3 pathways and modulation of gut microbiota.

### 4.3. AFB1 Induces Mitochondrial Dysfunction and Mitochondrial Apoptotic Pathway and the Improvement of Curcumin

Mitochondria are the principal producers of cellular adenosine triphosphate and the hub of energy metabolism in mammalian cells (ATP) [134]. Mitochondrial dysfunction is immensely related to the production of excessive ROS, which results in damage to cellular lipids, proteins, and other biomacromolecules, finally inducing cell apoptosis or necrosis [55,135]. Mitochondrial dysfunction had been illustrated as a critical mechanism of AFB1-induced cytotoxicity and tissue damage [136]. Significantly decreased mitochondrial membrane potential of cells as a major consequence of AFB1 exposure has been shown in several in vitro and animal studies [137,138]. Mitochondrial membrane potential generates the proton gradient, which is necessary in the process of ATP synthesis [139]. The activities of mitochondrial ETC I, III, and IV could control the mitochondrial membrane potential. Wan et al. discovered that broilers fed with the AFB1 diet had significant mitochondrial enlargement, loss of ATP levels, and reductions in the activity of (ETC) complexes I, II, III, and V in liver tissues [140]. Similar results were also found in a mouse model by Xu and colleagues [137]. AFB1 exposure also decreased transcription factor A (TFAM), nuclear respiratory factor 1 (Nrf1), dynamin-related protein 1(DRP1), fission protein 1 (FIS1), mitofusin 1 (MFN1), peroxisome proliferator-activated receptor-gamma (PPARγ) coactivator-1α (PGC-1α), and OPA1 mitochondrial dynamin-like GTPase (OPA1) expression, finally resulting in the inhibition of mitochondrial biogenesis and mitochondrial dynamics [136]. Another study also showed that AFB1 exposure could damage the mitochondrial ultrastructure and inhibit mitochondrial biogenesis and oxidative phosphorylation (OXPHOS)-mediated ATP production, followed by induction of mitochondrial dysfunction in the liver of mice [137]. Furthermore, AFB1 exposure may reduce the activity of the important tricarboxylic acid (TCA) cycle-rate-limiting enzymes succinic dehydrogenase, isocitrate dehydrogenase, and -ketoglutarate dehydrogenase [137]. Taken together, the action mechanisms of AFB1-exposure-induced mitochondrial dysfunction may include direct damage to the mitochondrial ultrastructure, a decrease in mitochondrial membrane potential, inhibition of TCA cycle-rate-limiting enzymes and mitochondrial biogenesis, loss of ETC complex activities, and a decrease in ATP levels.

Apoptosis is a type of programed cell death [141]. Apoptosis could be triggered by multiple exogenous stimuli, including bio-toxins, drugs, heavy metals, ultraviolet radiation, and pathogenic microorganisms [102,142]. It is generally known that in mammalian cells, mitochondrial malfunction causes the activation of the mitochondrial apoptotic pathway, which in turn causes caspase-dependent apoptosis [137]. The initiation of the mitochondrial apoptotic pathway is usually dependent on proteins of the Bcl-2 family, which mainly controls the permeabilization of the outer mitochondrial membrane by balancing between proapoptotic (e.g., Bcl-2-associated X protein (Bax) and Bcl-2 antagonist/killer-1 (Bak)) and antiapoptotic (e.g., Bcl-2, Bcl-2-like 2 (Bcl-w), myeloid cell leukemia-1 (Mcl-1), Bcl-2-like 1(Bcl-XL), and Bcl-2-related protein A1(Bfl-1)) expression [143]. The formation of mitochondrial outer membrane permeabilization could facilitate the release of CytC from the mitochondria to the cytoplasm in a cascade to activate caspases-9, -3 and cleaved PARP1, finally causing apoptotic cell death [144]. Some investigations found that AFB1 exposure increased Bax protein expression while decreasing Bcl-2 protein expression, resulting in the release of the CytC form from the mitochondria, the activation of caspases-9 and -3, and cell apoptosis [40,47,70,89,90,95,137,145]. In addition, AFB1 exposure could also trigger other signaling pathways, including p53, MAPKs, and Akt pathways, which play a critical role in regulating the mitochondrial apoptotic pathway [146,147].

It has been reported that curcumin administration could protect cells against mitochondrial damage via preventing mitochondrial membrane potential reduction and decreasing mitochondrial ROS production [55,56]. Li et al. demonstrated that curcumin supplementation could significantly improve AFB1-induced decrease in ATPases (including Mg^2+^-ATPase, Na^+^-K^+^-ATPase, Ca^2+^-ATPase, and Ca^2+^-Mg^2+^-ATPase activities), indicating that mitochondrial energy metabolism may be a critical target of curcumin’s protection [34]. Curcumin could suppress cell apoptosis by re-balancing the ratios between proapoptotic and antiapoptotic proteins. It has been reported that curcumin supplementation could reduce the Bax/Bcl-2 ratio, limit the development of mitochondrial outer membrane permeabilization, decrease the release of CytC, and lastly, ameliorate cell apoptosis caused by exogenous or endogenous stimulus [24,53,54,57,59,62,66,148]. Wang et al. found that curcumin supplementation could significantly improve mitochondrial dysfunction and apoptosis by downregulating the expression of CytC, Bax caspase-3, and caspase-9 mRNAs and proteins, and upregulating the expression of Bcl-2 mRNA and protein, thus improving the nephrotoxicity induced by AFB1 in a mouse model [70]. In addition, it was also reported that curcumin could significantly inhibit the expression of p53 and improve AFB1-induced mitochondrial dysfunction and mitochondrial apoptotic pathway in the liver tissues of chicken [81,82]. Consistently, El-Mekkawy et al. found that curcumin supplementation at 200 mg/kg BW for 90 days could significantly reduce the expression of p53 protein and increase the expression of Bcl-2 protein, thus inhibiting AFB1-induced apoptosis in the liver and kidney tissues of rats [35].

**Figure 4:**
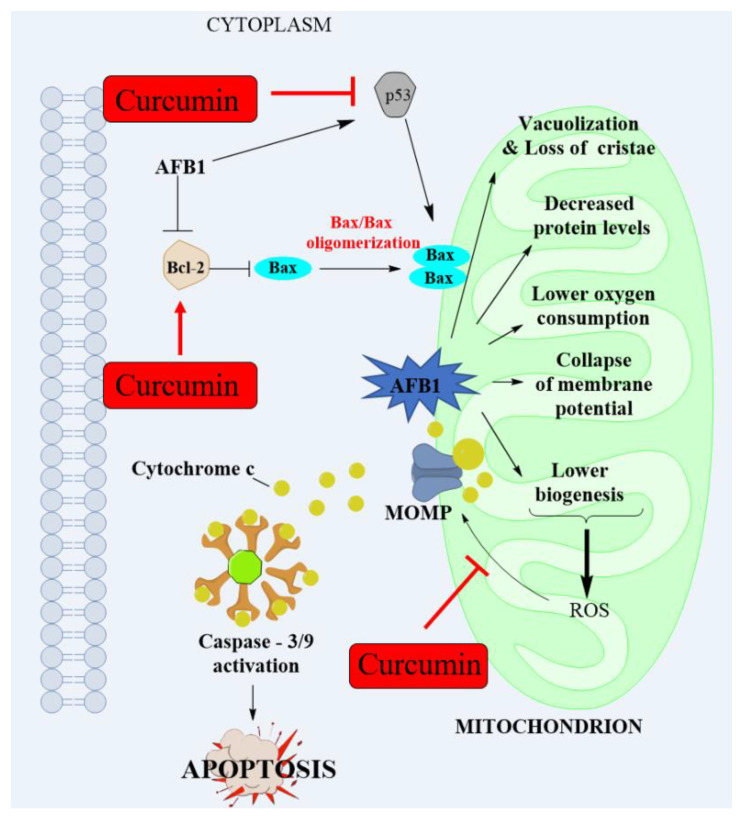
AFB1 exposure induces mitochondrial dysfunction and mitochondrial apoptosis and the modulated effects of curcumin.

Finally, AFB1 exposure could cause mitochondrial dysfunction and apoptosis via multiple molecules, including mitochondrial membrane potential, mitochondrial proteins, the activities of the complex in ETC and limiting enzymes in TCA, and the expression of Bcl-2, p53, Bax, CytC, caspases-9, and -3 proteins. Curcumin has the potential to target these sites and drastically ameliorate AFB1-induced toxic effects in mammalian cells. Furthermore, the molecular mechanism of AFB1-induced mitochondrial dysfunction and mitochondrial apoptotic pathway and the protective effects of curcumin are summarized in Figure 4.

### 4.4. AFB1 Induces Autophagy and Mitophagy and the Regulation of Curcumin

Autophagy is a multifaceted process. It might restore cellular equilibrium by destroying senescent or dysfunctional organelles and restoring nutrients. It has been demonstrated that autophagy dysfunction is relative with many diseases, including cancers, infective diseases, neurodegenerative diseases, aging, and immune dysfunction [149]. Under various stress conditions, including hypoxia, nutrient deficiency, DNA damage, and toxin exposures, autophagy could be activated and play a protective role [150]. Recent studies showed that AFB1 exposure could induce or inhibit autophagy, which is dependent on the toxic dose of AFB1 or organ exposure [47,151,152,153]. Huang et al. found that oral administration of AFB1 at the doses of 0.375, 0.75, or 1.5 mg/kg body weight for 30 days significantly upregulated the expression of autophagy-related proteins LC3, Beclin1, Atg5, and p62 expression, and it downregulated the expression of PI3K, p-AKT, and p-mTOR in the testicular tissue, finally resulting in spermatogenesis in male mice [47]. Xu et al. found that AFB1 exposure (at 40 μM) could upregulate autophagy via the inhibition of the EGFR/PI3K/mTOR pathway in L02 cells, and rapamycin significantly inhibited AFB1-induced apoptosis via the autophagy induction [108]. Similarly, AFB1 could upregulate autophagy and autophagy flux in RAW264.7 cells, which is partly attributed to the production of ROS and the activation of the ERK pathway. Interestingly, the downregulation of AFB1-induced autophagy by ATG7 knockdown significantly abolished AFB1-induced inflammatory response [152]. On the contrary, Chen et al. found that AFB1 exposure (at 10 μM for 24 h) significantly inhibited AMPK/mTOR-mediated autophagy flux, thus promoting the production of ROS in a cascade to induce apoptotic cell death in Leydig cells in vitro [154].

Mitophagy, a specialized form of autophagy, could remove dysfunctional or superfluous mitochondria and maintain the mitochondria homeostasis [155]. In eukaryotic cells, the PTEN-induced putative kinase 1 (PINK1)/E3 ubiquitin ligase PARK2 (Parkin) pathway is the most comprehensively characterized pathway of mitophagy [155]. PINK1 could accumulate on the mitochondrial membrane and induce Parkin recruitment to facilitate the degradation of damaged mitochondria [155]. AFB1 exposure has been shown to dramatically enhance the expression of PINK1 and Parkin proteins in mouse liver, kidney, and spleen tissues [156,157,158]. Furthermore, Parkin deficiency could significantly aggravate AFB1-induced mitochondrial damage and oxidative stress by blocking mitophagy, thus promoting AFB1-induced liver, kidney, and spleen injuries [156,157,158]. These data indicated that mitophagy may play a protective role during AFB1-exposure-induced adverse effects in animals or humans. Additionally, a previous study found that AFB1 exposure at a dose of 0.6 mg/kg for 28 days could significantly alter the expression of PINK1, Parkin, and COX-2, and the knockout of COX-2 significantly inhibited AFB1-induced mitophagy [159]. COX-2 also play critical roles in a variety of pathological processes, including cell proliferation, ferroptosis, inflammatory reaction, and apoptosis [159]. It indicated that COX-2 may play an important role in AFB1-induced mitophagy. It also provided new evidence for the crosstalk between mitophagy and other processes (i.e., cell proliferation, ferroptosis, inflammatory reaction, and apoptosis). More investigations into this key molecular mechanism are required.

**Figure 5:**
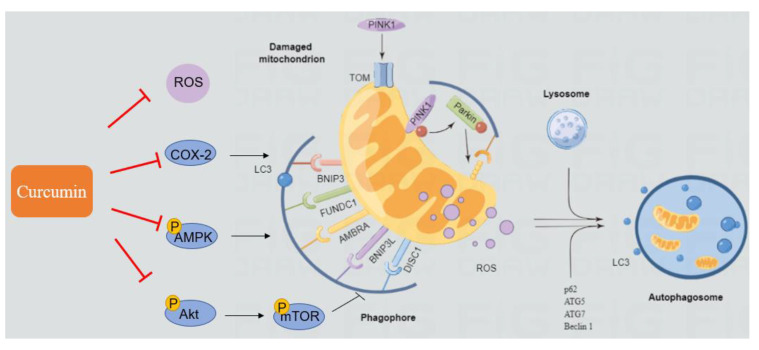
The regulatory effects of curcumin on AFB1-induced autophagy or mitophagy.

Many studies have revealed that curcumin can activate autophagy, although the molecular mechanisms are different [160,161]. Curcumin could induce autophagy via the downregulation of the PI3K/Akt/mTOR pathway, induction of AMPK and extracellular signal-regulated kinases 1 and 2 (ERK1/2) pathways, induction of ROS formation, and promotion of ER stress [160,161]. Muhammad et al. showed that curcumin supplementation could upregulate the expression of Beclin1, ATG5, Dynein, and LC3A proteins, and downregulate the expressions of p53 and mTOR proteins, thus conferring hepatoprotection against AFB1-induced toxicity in chicken [82]. Consistently, it was also reported that curcumin supplementation could upregulate Parkin-mediated mitophagy via the activation of the AMPK pathway and could protect against oxidative-stress-induced intestinal barrier injury [162]. In addition, curcumin is also an effective inhibitor of COX-2 and ROS, which are two positive players in the processes of AFB1-induced autophagy. Therefore, curcumin could inhibit AFB1-induced autophagy or mitophagy by directly inhibiting the production of ROS and the expression of COX-2. There is evidence indicating that curcumin supplementation may provide a protective effect by modulating autophagy and mitophagy. A diagram depicting the modulated effects of curcumin on AFB1-induced autophagy or mitophagy is shown in Figure 5. Further research into animal models and detailed molecular processes is required.

### 4.5. The Bioactivation and Detoxification of AFB1 and Regulation of Curcumin

Coincidentally, the liver is not only the main target organ of AFB1 but also the main site of AFB1 metabolism [9]. Multiple metabolic enzymes, including CYP450s, epoxide-hydrolases, aldehyde-reductases, monooxygenases, amino-oxidases, alcohol dehydrogenases, and ketone-reductases, were demonstrated to participate in the metabolism of AFB1 [163,164]. It is well illustrated that AFB1 could be metabolized via two-stage reactions in the liver. The first-stage metabolisms of AFB1 in the liver tissue include the reduction reaction (ketoreduction to AFL), oxidative reaction (O-dealkylation to AFP1), hydrolytic reactions (hydroxylation to AFM1, AFQ1, and AFB2), and this process involves various enzymes, including CYP450, monooxygenases, amino-oxidases, alcohol dehydrogenases, epoxide-hydrolases, aldehyde-reductases, and ketone-reductases [165,166,167]. AFTs are primarily regulated by the crucial metabolic enzymes CYP450s [168]. Studies have shown that CYP3A37, CYP3A4, CYP2A13, CYP2A6, CYP1A5, CYP1A2, and CYP1A1 are mainly responsible for the biological conversion of AFB1 into AFBO in liver tissues [169,170,171]. A recent study also found that CYP450 enzymes were also detected in intestinal tissues, which also contributed to the toxic effect of AFB1 in intestinal tissues [26]. The second-stage reaction of AFB1 metabolism mainly involved a covalent binding reaction and a conjugation reaction, which resulted in two different endpoints, i.e., enhancing toxic effect, and excretion and detoxification, respectively [172]. For example, AFBO, one of AFB1’s primary metabolites, might react with DNA, thus inducing toxicity [172]. In contrast, glutathione-S-transferase (GST) M1 and GSTT1-derived conjugation of GSH to AFBO contributes to the detoxification of AFB1 [167].

Curcumin has been shown to regulate AFB1 metabolism and detoxification through influencing the activity and mRNA expression of CYP450 enzymes [173]. Multiple studies have demonstrated that curcumin exhibits potent regulatory abilities on the activities and expressions of CYP450 enzymes, and it exhibits potent therapeutic effects on nonalcoholic fatty liver disease (NAFLD), cardiovascular diseases, and exogenous-compounds-induced toxicity [174]. Previous research found that curcumin could limit the biotransformation of AFB1 into AFBO and other aflatoxin metabolites by inhibiting CYP450 isoenzymes, particularly CYP2A6 subtypes, reducing the adducts formed by AFBO with DNA and protein, and thus reducing the toxicity and carcinogenicity of AFB1 in cells [25]. In another study, Zhang et al. found that curcumin supplementation could inhibit the expression of CYP1A1, CYP1A2, CYP2A6, and CYP3A4 in the liver tissues of broilers, followed by reducing the formation of AFBO transformed from AFB1 [28,30,175]. Curcumin supplementation was observed to downregulate the mRNA expression of CYP3A28, which therefore reduced the synthesis of AFM1 transformed from AFB1, ultimately leading to curcumin’s protective impact against AFB1-induced cytotoxicity in BFH12 cells [60]. It was also found that curcumin supplementation could induce the expression of P-gp protein in the small intestine tissue of chicken, which resulted in a decrease in uptake of AFB1 and reduced its toxicity [26].

Furthermore, as previously stated, curcumin may directly trigger the transcriptional activation of Nrf2, therefore promoting the production of several genes of CYP450 enzymes or phase II enzymes that guide the first and second stages of AFB1 metabolism [32]. Indeed, it had been demonstrated that the activation of Nrf2 could be positively relative to the expression of CYP1A2, CYP2A5, CYP2C29, CYP2E1, and CYP2B10 in the liver tissues of mice [176]. Muhammad et al. demonstrated that curcumin supplementation could upregulate the expression of Nrf2 and its downstream genes, such as GSTA3 and GSTM2, in the liver tissues of broilers, thus improving AFB1-induced liver toxicity via the GSTA3- and GSTM2-mediated detoxification effects [32].

**Figure 6:**
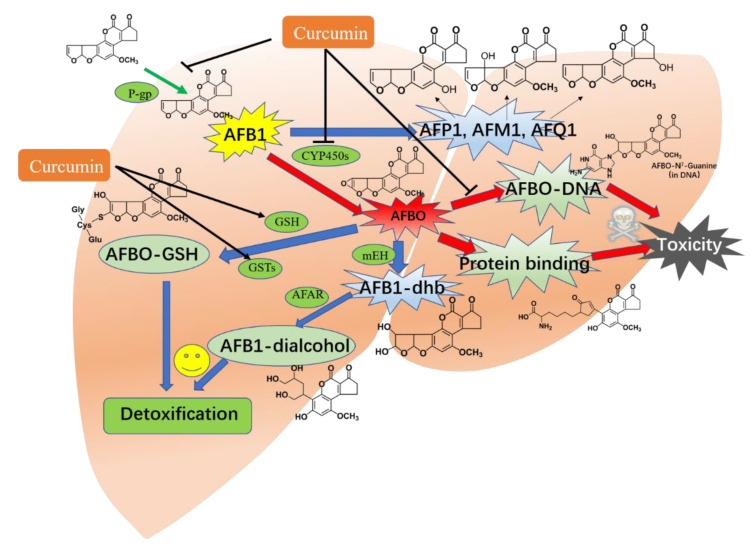
Overview of the metabolism pathways of AFB1 and the modulation of curcumin. Curcumin could regulate the AFB1 uptake and intracellular metabolism by regulating the expression of P-gp and GSTs proteins and the activities of CYP450s and the formation of AFBO-DNA adduct. AFB1, aflatoxin B1; aflatoxicol M1; aflatoxin P1; AFQ1, aflatoxin Q1; AFB1-dhd, AFB1-8,9-dihydrodiol; AFAR, aflatoxin-aldehyde reductase; AFBO, exo-AFB1-8,9-epoxide; GSTs, glutathione S-transferases; GSH, glutathione; mEH, microsomal epoxide hydrolase; CYP450s, cytochrome P450s; P-gp, P-glycoprotein. This figure is modified based on the original figure published in Deng et al. [167].

In conclusion, phase I and II metabolizing enzymes, such as CYP450s, GST isozymes, and others might govern the bioactivation and detoxification of AFB1, which are essential steps in the process of AFB1-caused toxicity. Some variables, including species, age, environment, and health state, may influence AFB1 toxicity via altering the first- and second-stage metabolisms. Indeed, the regulatory effects of curcumin on the first- or second-stage metabolisms of AFB1 involve the regulation of the activities or expressions of CYP450s (i.e., CYP1A1 and CYP2E6), Nrf2-mediated II metabolizing enzymes or products (i.e., GST isozymes and GSH), and P-gp, which may be considered a critical molecular mechanism for explaining its protective effects on AFB1-induced toxicity. An overview of the metabolism pathways of AFB1 and the modulation of curcumin is shown in Figure 6.

## 5. Conclusions and Future Directions

**Figure 7:**
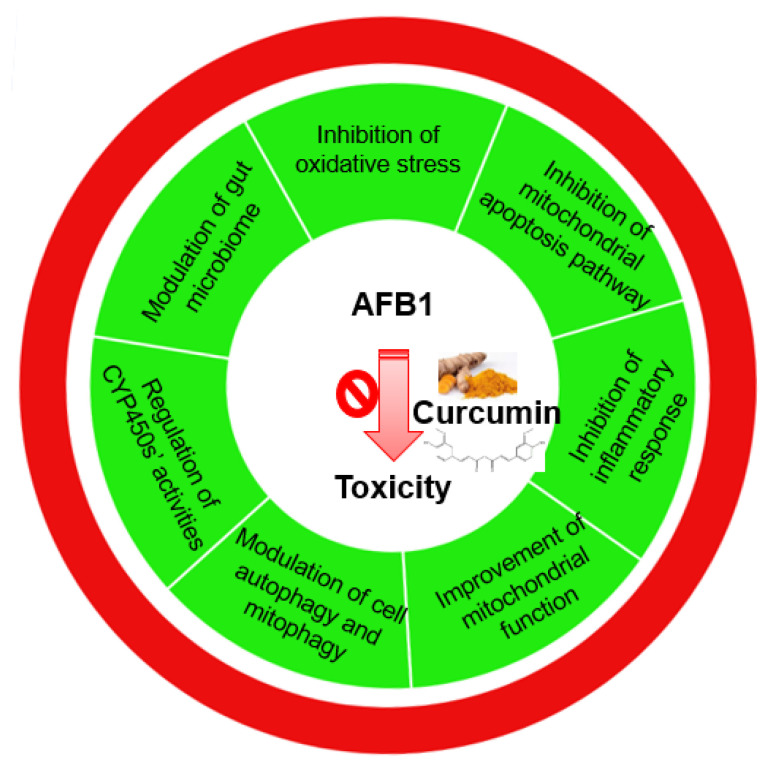
The main actional mechanisms of curcumin protection against AFB1 toxicity.

Due to toxicity and potent carcinogenicity, AFB is considered one of the most important mycotoxins among over 500 mycotoxins found worldwide. The molecular mechanisms of AFB1-induced basic effects are complex. In the past several decades, scientists have demonstrated that AFB1 could induce oxidative stress, lipid peroxidation, apoptosis, mitochondrial dysfunction, autophagy, necrosis, immune repression, and inflammatory response, finally resulting in various acute or chronic diseases in animals and humans, including cancer, hepatitis, mutation abnormalities, and reproduction disorders. The critical signaling pathways involved are the p53, mTOR, Nrf2, NF-κB, NLRP3, MAPKs, and Wnt/β-catenin pathways. These advancements provided essential goals for the investigation and development of efficient improvement strategies to counteract the unintended negative effects caused by consuming diet food or feed contaminated with AFB1 in humans or animals. Over the last two decades, researchers have made significant progress in the creation of effective nutritional or natural product supplements, which can effectively ameliorate AFB1-contamination-caused harm to human and animal health. Curcumin exhibited potently protective effects against AFB1-induced cytotoxicity, hepatotoxicity, and nephrotoxicity in vitro and in vivo. Importantly, human clinical trials and animal trials reveal that curcumin has high safety at a cheap price. The molecular mechanism of curcumin’s protection comprised the inactivation of CYP450 enzymes, prevention of mitochondrial malfunction and apoptosis, free radical scavenging, downregulation of oxidative stress and inflammatory response, modification of autophagy, and gut microbiome (Figure 7). However, the primary barriers to curcumin use in clinics continue to be its limited bioavailability, poor solubility, and quick disintegration in human or animal bodies. Furthermore, existing evidence of curcumin-mediated detoxifying activities against AFB1-induced toxicity comes mostly from animal investigations and in vitro cell models, whereas successful clinical trials are sparse. As a result, considerably more research is required.

## Figures and Tables

**Table 1:** A summary of in vitro and animal models involving the protective effects of curcumin or curcuminoids on AFB1-induced toxicity.

Cells/Animals	Treatments	Regulated Effects of Curcumin	References
Bovine SV40 large T-antigen-transduced fetal hepatocyte-derived cell line BFH12	BFH12 cells were pretreated with an aryl hydrocarbon receptor (AHR) agonist (i.e., PCB126) at 1 nM for 24 h; then, cells were pretreated with curcumin (purity ≥94%) and Curcuma longa extracts (purity ≥80% curcumin) for 16 h, then co-treated with AFB1 (3.6 μM) for further 48 h.	Curcumin and Curcuma longa extracts both exhibited protective effects against AFB1-induced cytotoxicity in BFH12 cells. The main molecular pathways involved antioxidant and anti-inflammatory response, cancer, and drug metabolism.	[77]
5-week-old male BALB/c mice	Mice were orally administrated with curcumin at 100 or 200 mg/kg BW with or without AFB1 at 0.75 mg /kg BW for 30 days. After treatment, liver tissues were collected for assessment.	Curcumin reduced the accumulation of AFB1-DNA adducts in the liver and alleviated hepatotoxicity by inhibiting AFB1-induced oxidative stress and potentiating GST-mediated phase II detoxification. Curcumin inhibited AFB1-induced pyroptosis via inhibiting the activation of NLRP3-mediated inflammasome. It also inhibited AFB1-induced inflammatory response and oxidative stress via upregulating the Nrf2 pathway.	[71]
Male rats (BW is in the range of 100 ± 5 g)	Rats were intraperitoneally injected with AFB1 at one dose of 3 mg/kg BW; then, rats were orally treated with curcumin at 15 mg/kg for 5 weeks. Finally, the liver tissues were collected.	Curcumin treatment exhibited a good therapeutic effect. Curcumin treatment significantly upregulated the activities and mRNA expression of antioxidant enzymes CAT, SOD, and GPX, GST, and it upregulated the levels of GSH in the liver tissues of rats.	[33]
*Nile tilapia Oreochromis niloticus*	Oreochromis niloticus were injected with 6 mg/kg BW; then, they were fed with curcumin at 10 or 20 g/kg (all fish were fed twice daily at a feeding rate of 3% of the actual BW). After 14 days, the liver, kidney, and blood were collected.	Curcumin supplementation could significantly improve AFB1-induced liver and kidney damage. Meanwhile, curcumin supplementation could significantly upregulate the expression of antioxidant gene in the liver tissues of *Oreochromis niloticus*.	[78]
18-day-old male broiler chicken	Chicken was fed with curcumin at a dose of 400 mg/kg with or without AFB at a dose of 0.02 mg/kg for 10 days.	Curcumin supplementation significantly improved AFB1-induced lipid peroxidation, DNA damage, and oxidative stress. Meanwhile, curcumin significantly inhibited the expression of NADPH Oxidase 4 (NOX4) mRNA and protein.	[75]
One-day-old commercial Arbor Acres (AA) broilers	Birds were fed with 150, 300, and 450 mg curcumin (purity = 2.5%)/kg feed with or without AFB1 (purity ≥99.0%) at 5 mg/kg feed for 28 d, respectively. Finally, the liver, kidney, and muscle tissue samples were collected.	(1) Curcumin supplementation significantly alleviated AFB1-induced toxicity and oxidative stress by inhibiting the generation of ROS, 8-OHdG, the formation of AFB1 adducts, the expression of mitochondrial apoptosis-related genes, the activities of CYP2A6 enzyme, and activating the Nrf2 signaling pathway in the liver tissues. (2) Curcumin also increased AFB1-GSH conjugation in vitro in liver cytosol. (3) Curcumin supplementation in the diet reduced the clearance time of AFM1 in liver and kidney but not in muscle tissues.	[79,80,81,82,83,84]
One-day-old ducks (*Anas platyrhynchos*)	Ducks were fed with 500 mg curcumin /kg feed for 70 days; then, they were orally exposed to AFB1 at 60 μg/kg BW. After 12 h, the blood and liver samples were collected.	Curcumin supplementation in the diet could significantly inhibit the generation of H_2_O_2_, MDA, and the formation of AFB1-DNA, and it could activate the Nrf2-ARE signaling pathway and suppress the NLRP3/caspase-1 and NF-κB signaling pathways in the liver and ileum tissues of ducks.	[26,69]
One-day-old ducklings	Ducklings were fed with 400 mg/kg curcumin-containing feed with or without AFB1 at 0.1 mg/kg BW (intragastric administration) for 21 days. The spleen tissues and serum samples were collected.	Curcumin supplementation upregulated the Nrf2 signaling pathway and the expression of related antioxidant enzymes, and it inhibited the NF-κB signaling pathway and reduced the expression of related inflammatory factors, finally improving AFB1-induced spleen tissue damage.	[72,79]
5-week-old male Fischer rat	Rats were fed with curcumin at doses of 8 or 80 mg/kg BW with or without AFB at a dose of 0.1 mg/kg BW for 3 consecutive weeks (5 days in each week). Finally, the blood and liver samples were collected.	Curcumin supplementation significantly improved the liver function. Curcumin also reduced glutathione S-transferase (GST) placental form positive single cells and foci caused by AFB1 treatment.	[74,85]
One-day-old Arbor Acres (AA) broilers	Broilers were fed with curcumin at 300 mg/kg with or without AFB1 1 mg/kg for 28 d. Liver samples were harvested.	Curcumin partially attenuated the abnormal morphological changes, oxidative stress, and apoptosis in liver tissues.	[73]
Sprague Dawley rats	Rats were fed with curcumin (purity ≥98.0%) at 200 mg/kg BW with or without AFB1 (purity ≥99.0%) at 25 µg/kg BW (orally given) for 90 days. The liver and kidney samples were collected.	Curcumin improved AFB1-induced inflammatory response and oxidative stress in the liver and kidney tissues of rats. Meanwhile, curcumin reduced the expression of p53 protein and increased the expression of Bcl-2 protein, thus inhibiting AFB1-induced apoptosis in the liver and kidney tissues of rats.	[35,72]
Non-cancerous (HUC-PC) urinary bladder cells	Curcumin pretreatment at 1.56 μg/mL, then co-treated with AFB1 at the final concentration of 5 μM for additional 24 h.	Curcumin pretreatment exhibited cytoprotective effects by ameliorating AFB1-induced cytotoxicity with inferred tendencies to prevent carcinogenesis.	[85,86]
5-week-old male BALB/c mice	Mice were administrated with curcumin at doses of 100 and 200 mg/kg BW, then co-treated with AFB1 at a dose of 750 μg/kg BW for 30 days.	Curcumin supplementation significantly inhibited AFB1-induced renal oxidative stress and apoptosis via the inhibition of mitochondrial apoptotic pathway (downregulating the expression of CytC, Bax, cleaved-Caspase-3, Caspase-9 proteins and upregulating the expression of Bcl-2 mRNA and protein) and the activation of Nrf2 pathway (i.e., upregulating the expression of CAT, SOD1, NQO1, GSS, GCLC, and GCLM mRNAs and proteins).	[70,71]
Three-month-old male Sprague Dawley rats	Rats were treated with curcumin nanoparticle loaded hydrogels at doses of 100 or 200 mg/kg BW, then orally treated with or without AFB1 at a dose of 0.125 mg/kg BW for 3 weeks. Blood and liver samples were collected.	Curcumin nanoparticle loaded hydrogels at 100 or 200 mg/kg BW could significantly improve AFB1-induced fibrosis, inflammatory response, genotoxicity, and apoptosis in the liver tissues of rats.	[31]
*Nile tilapia Oreochromis niloticus*	Fish fed with 200 ppb of AFB1 with and without curcumin at 5 mg/kg for 16 weeks.	Fish fed with AFB1-contaminated diet showed an upregulation of CYP1A and downregulation of SOD, IL-1β, and TGF-β in the liver tissues, which were effectively revised by curcumin supplementation.	[87]
One-day-old Arbor Acres broilers	Birds were fed with 150, 300, and 450 mg curcumin (purity ≥2.5%)/kg feed with or without AFB1 at 5 mg/kg feed for 28 d, respectively. Finally, the duodenum tissues were isolated for further examinations.	Curcumin supplementation could ameliorate AFB1-induced duodenal toxicity and damage through downregulating CYP450 enzymes, promoting ATPase activities, and inducing the expression of p-glycoprotein (P-gp).	[26,78]
16-week-old male Fisher—344 rats	Rats were fed with AFB1 at 20 μg/day for 6 weeks and co-treated with dietary curcumin (0.05%, *w/w*) for 3 weeks.	Curcumin supplementation significantly improved AFB1-induced liver dysfunction, upregulated the GSHT and UGT1A1 activities, and downregulated the activity of CYP1A1. Curcumin supplementation significantly reduced AFB1–N7-guanine adduct (*p* < 0.001) excretion in the urine, DNA adduct in the liver, and albumin adduct in the serum.	[30,70]

## Data Availability

Not applicable.
